# Automated Localization of Focal Ventricular Tachycardia From Simulated Implanted Device Electrograms: A Combined Physics–AI Approach

**DOI:** 10.3389/fphys.2021.682446

**Published:** 2021-07-01

**Authors:** Sofia Monaci, Karli Gillette, Esther Puyol-Antón, Ronak Rajani, Gernot Plank, Andrew King, Martin Bishop

**Affiliations:** ^1^King’s College London, London, United Kingdom; ^2^Division of Biophysics, Medical University of Graz, Graz, Austria

**Keywords:** ventricular tachycardia, implanted devices, electrograms, automated localization, torso modeling, deep learning

## Abstract

**Background:** Focal ventricular tachycardia (VT) is a life-threating arrhythmia, responsible for high morbidity rates and sudden cardiac death (SCD). Radiofrequency ablation is the only curative therapy against incessant VT; however, its success is dependent on accurate localization of its source, which is highly invasive and time-consuming.

**Objective:** The goal of our study is, as a proof of concept, to demonstrate the possibility of utilizing electrogram (EGM) recordings from cardiac implantable electronic devices (CIEDs). To achieve this, we utilize fast and accurate whole torso electrophysiological (EP) simulations in conjunction with convolutional neural networks (CNNs) to automate the localization of focal VTs using simulated EGMs.

**Materials and Methods:** A highly detailed 3D torso model was used to simulate ∼4000 focal VTs, evenly distributed across the left ventricle (LV), utilizing a rapid reaction-eikonal environment. Solutions were subsequently combined with lead field computations on the torso to derive accurate electrocardiograms (ECGs) and EGM traces, which were used as inputs to CNNs to localize focal sources. We compared the localization performance of a previously developed CNN architecture (Cartesian probability-based) with our novel CNN algorithm utilizing universal ventricular coordinates (UVCs).

**Results:** Implanted device EGMs successfully localized VT sources with localization error (8.74 mm) comparable to ECG-based localization (6.69 mm). Our novel UVC CNN architecture outperformed the existing Cartesian probability-based algorithm (errors = 4.06 mm and 8.07 mm for ECGs and EGMs, respectively). Overall, localization was relatively insensitive to noise and changes in body compositions; however, displacements in ECG electrodes and CIED leads caused performance to decrease (errors 16–25 mm).

**Conclusion:** EGM recordings from implanted devices may be used to successfully, and robustly, localize focal VT sources, and aid ablation planning.

## Introduction

Ventricular tachycardia (VT) is a serious cardiac arrhythmia that represents an important source of morbidity and, upon degeneration into more lethal arrhythmias such as ventricular fibrillation (VF) (Srinivasan and Schilling, 2018), sudden cardiac death (SCD) (Harris and Lysitsas, 2016; Ritchie and Roser, 2018). Hence, the prevention of VT, and its degeneration into VF, is of primary clinical importance to improve morbidity and reduce mortality.

In structurally healthy hearts, VT occurs primarily as a consequence of abnormal ectopic firing in the ventricles, overtaking sino-atrial activation and leading to premature ventricular contractions (PVCs). An effective treatment against ectopic VT is radiofrequency catheter ablation, which aims to target the tachycardia by first locating, and then electrically isolating the region causing the episode. However, procedure success is heavily dependent on an accurate localization of the VT source. Often, recordings of the focal VT, in the form of an electrocardiogram (ECG) or implanted device electrograms (EGM), exist prior to an ablation procedure, which inherently contain important information related to the focal origin of the VT source. Integration of computational studies and deep learning approaches provides an exciting opportunity to utilize the information contained within these recordings to potentially facilitate automated VT localization into clinical practice.

In recent decades, computational studies (Trayanova, 2011; Clayton and Bishop, 2014; Henriquez, 2014; Niederer et al., 2019; Yu et al., 2019) have enhanced greatly our knowledge of VT mechanisms and have strengthened diagnostic, therapeutic, and prognostic VT clinical strategies (Rantner et al., 2013; Trayanova et al., 2017; Mendonca Costa et al., 2019; Niederer et al., 2019), helping in the growth of personalized modeling (Prassl et al., 2009; Relan et al., 2011; Medtronic, 2016; Cedilnik et al., 2018; Le Bras, 2018; Potse, 2018). One limitation of the majority of these studies is the dependence on monodomain formulations to represent electric sources in the form of transmembrane voltages. These models are time-consuming, and thus to achieve clinical translation, fast reaction-eikonal (RE) simulations (Neic et al., 2017; Cedilnik et al., 2019) have been the preferred choice. More recently, realistic simulations of full extracellular potentials at specific locations (e.g., ECG electrodes) have been obtained from the combination of lead field (LF) methods (Potse, 2018) with fast RE models (Gillette et al., 2021), achieving accuracy comparable to pseudodomain or bidomain formulations, but within a fraction of the computational time.

Using computational simulations of electrophysiological (EP) behavior has also been exploited to provide training datasets for machine and deep learning algorithms (Yang et al., 2018; Shade et al., 2020); however, these studies did not utilize rapid RE models (Yang et al., 2018; Shade et al., 2020), or LF methods (Shade et al., 2020). Yang et al. (2018) were among the first to utilize convolutional neural networks (CNNs) to localize focal VT sources from simulated ECGs. The novelty of the study was in the integration of computational simulated data with CNN architectures; previous studies had in fact attempted to localize focal VTs from either simulated ECGs—utilizing myocardial activation imaging techniques—with no use of artificial intelligence (van Dam et al., 2009)—or clinical ECGs utilizing machine learning algorithms (Zhou et al., 2019).

One important limitation of Yang et al. (2018) was the restriction of the method to the use of ECGs. Although ECGs are widely used as a routine modality for VT management, they are not always available for VT patients, particularly focal VT patients in which the clinical VT is not inducible. Utilizing cardiac implantable electronic device (CIED) EGMs, which the majority of pre-ablation patients have *in situ* (Pekka Raatikainen et al., 2014; Winterfield et al., 2018), and which continuously record and store any abnormal arrhythmic activity, could bring great improvements to the automated localization of focal VT. Recent clinical studies have demonstrated that stored EGM recordings of re-entrant VT episodes from implanted devices can be successfully used to guide the construction of pace-maps during an ablation procedure, with similar accuracy to the use of ECGs, but with the advantage of ensuring that the clinical VT is targeted (Yoshida et al., 2010; Yokokawa et al., 2019). In our own recent work, we demonstrated how such EGM recordings might be utilized to perform patient-specific *in silico* pace-mapping (Monaci et al., 2020), improving pre-procedural ablation planning for complex scar-related VTs. However, literature lacks further investigation on the power of EGM recordings for the localization of focal VTs, for which computational models can address and answer a variety of different questions, and their use in AI-based algorithms.

In this study, we demonstrate the utility of leveraging the information contained within simulated implanted device EGM recordings for the automated localization of focal VTs in the LV. This could benefit clinical procedures by providing pre-procedural ablation information of the VT episodes without the necessity of acquiring ECG recordings of the focal VT, which represents the long-term aim of our study. Although the majority of idiopathic VTs originate in the right ventricular outflow track (RVOT) (Srivathsan et al., 2005), focal VTs can also originate from a variety of different locations in the LV (Ito et al., 2003; Srivathsan et al., 2005; Yamada et al., 2008), and automating their localization could be beneficial to clinical procedures. To achieve our goal, we extend the previous work of Yang et al. (2018) and utilize fast computational simulations (RE combined with LF) on a realistic image-based torso model to generate ECG and EGM traces, which serve as inputs to a CNN architecture. We show the possibility of obtaining comparable localization in Cartesian coordinates between ECG-based and EGM-based trained CNNs. Moreover, we show improvement in the overall localization by introducing a novel CNN algorithm, utilizing a local ventricular-specific coordinate system (Bayer et al., 2018).

## Materials and Methods

**FIGURE 1:**
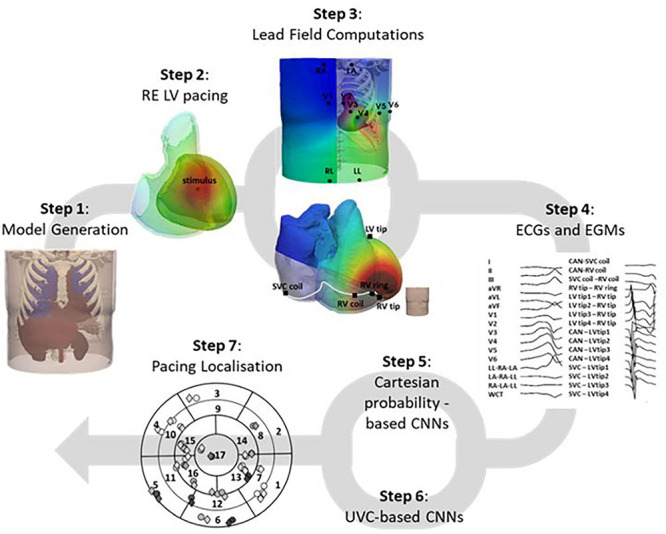
Study workflow. The 3D torso model, generated (step 1) from a CT TAVI planning scan, was utilized to pace the LV from different locations, within a fast RE environment (step 2). These solutions were combined with LF matrices computed (step 3) on the 12-lead ECG electrodes and manufacture-guided CIED leads to generate accurate ECG and EGM traces (step 4), which were then used as inputs to two CNN architectures, one Cartesian probability-based (step 5), and one novel UVC-based (step 6). Localization of the paced beats across the LV was then computed (step 7) and compared to the actual locations of the simulated beats.

The workflow of this study is summarized in Figure 1. Briefly, this involved using a previously generated 3D torso model (Monaci et al., 2020) (step 1) to rapidly simulate focal pacing across the LV within a RE environment (step 2). These simulated paced beats were combined with LF matrices computed on the standard 12-lead ECG electrodes and manufacturer-guided CIED right (RV) and left (LV) ventricular leads (step 3) to reproduce accurate 12-lead ECG and EGM recordings of the paced beats (step 4). The data were then processed and used as input to an adapted version of a previously developed CNN architecture by Yang et al. (2018) (step 5) and also to a novel network, consisting of a two-output regression and a classification CNN (step 6), and utilizing UVCs, to localize the paced beats (step 7).

### Model Preparation

As in Monaci et al. (2020), a 3D torso model was generated from a computed tomography (CT) trans-catheter aortic valve implantation (TAVI) panning scan. The torso model included all major organs, with conductivities reported in Table 1, and a detailed four-chamber heart, extracted from a separate cardiac CT scan. The patient did not present any visible structural heart disease and consented to the use of their data in ethically approved research: UK Research Ethics Committee reference number 19/HRA/0502 and 15/LO/1803. To decrease computational time without a loss of physiological electric signals, the average ventricular edge length of the biventricular mesh was kept at 738 μm. Realistic fiber orientation was incorporated into the ventricular myocardium using a well-established rule-based approach (Bayer et al., 2012).

**TABLE 1:** Organ conductivities of our torso model.

**Organs**	**Tissue conductivities (S/m)**
Lungs	0.0714
Bones	0.05
Skin	0.05
Fat/Muscle	0.24725
Liver	0.1667
Spleen	0.1
Kidneys	0.1667
Aorta	0.6667
Ventricular blood pools	0.6667
Atrial blood pools and walls	0.6667
Pericardium	0.2

*See also Plancke et al. (2019).*

**FIGURE 2:**
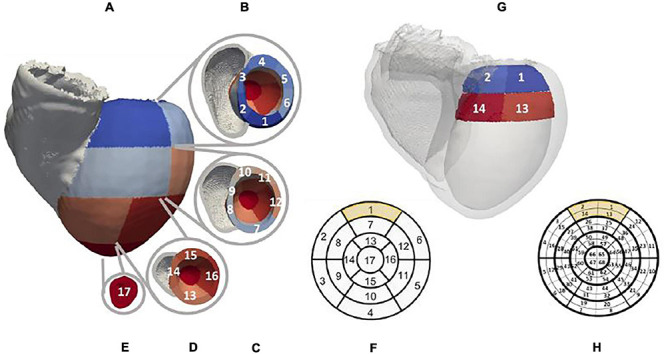
Patient-specific LV segment models. Generic AHA 17-segment model is shown in **(F)**. The equivalent patient-specific model of the LV mesh is shown in **(A)** with basal, mid, and apical segments illustrated in **(B–E)**, respectively. An example of the novel 68-segment model is shown in **(H)** highlighting the equal division in four parts of each of the 17 segments. **(G)** shows an example of how segment 1 in our mesh was divided into four equal segments.

The LV was geometrically divided into 17 segments, according to the American Heart Association (AHA) guidelines (Selvadurai et al., 2018), as shown in Figures 2A–F. In addition, each of the segments was subsequently divided into four, for a total of 68 (Figures 2G,H). These models were used as guidance for the collection of pacing locations, for the generation of training and testing labels for the existing CNNs, and the visualization of the localized VT sources.

To replicate focal ectopic VTs across the LV segments, ∼3767 randomly chosen paced beats—single stimuli, with a basic cycle length (BCL) of 400 ms—were simulated using a computationally efficient RE formulation (Neic et al., 2017) within the Cardiac Arrhythmia Research Package (CARP) (Vigmond et al., 2003), utilizing the 10 Tusscher ventricular cell model (ten Tusscher et al., 2004). Intra- and extracellular tissue conductivities were tuned to achieve physiological QRSs (Costa et al., 2013), comparable to equivalent pseudo bidomain simulations on a higher-resolution mesh (Monaci et al., 2020). Intra- and extracellular conductivities were 0.1845S/m and 0.6628S/m along the fiber direction, respectively, and 0.0493S/m and 0.1769S/m transverse to it. The corresponding RE conduction velocities (CVs) were 0.5455m/s and 0.1802m/s, along and transverse the fiber direction, respectively.

**FIGURE 3:**
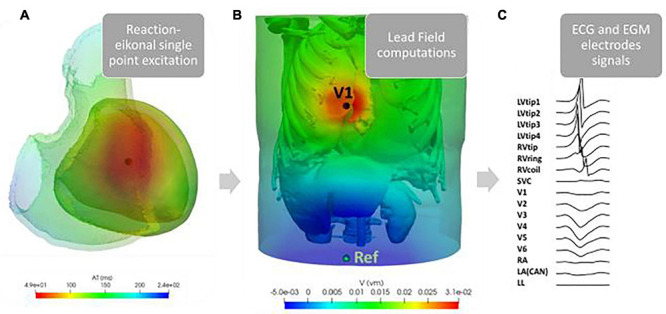
Example of our simulation pipeline. RE single point excitations were simulated in ∼3767 different locations across the LV [an example of the activation time map generated for a single such paced beat is shown here in **(A)**]. These solutions were combined with the LF matrices computed on the standard nine ECG leads (V1–V6, RA, LA, and LL), and nine EGM sensing points (LVtip1–4, RVtip, RVring, RVcoil, SVC, and CAN = LA), here shown in **(B)** for LF on V1. The final signals at each lead, shown in **(C)**, were then combined to obtain vector combinations shown in Figures 4E,F.

**FIGURE 4:**
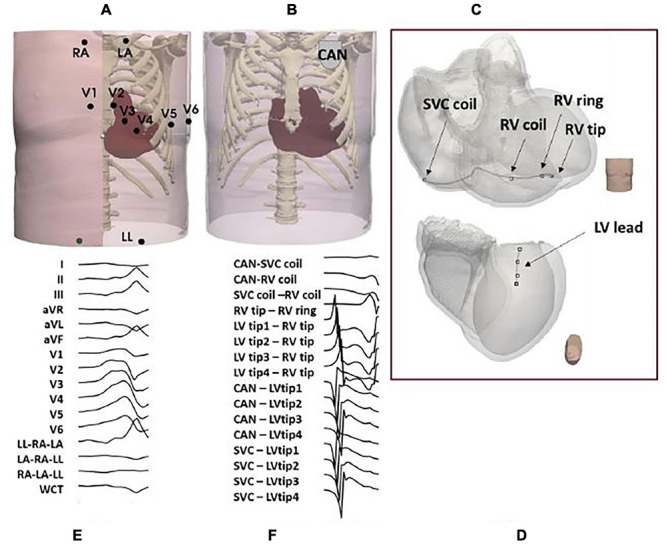
Torso setup. ECG and CIED configurations are shown in **(A–D)**. Example of 16 combinations of pacing signals used for training and testing are illustrated in **(E)** for ECGs and **(F)** for EGMs.

To allow the computation of extracellular potential signals from specific locations within the torso (Figure 3), the simulated cardiac potentials of each paced beat were combined with the LF Method (Potse, 2018). Specifically, LF matrices were calculated within CARP (Vigmond et al., 2003) on the standard ECG lead locations and on the RV and LV lead sensing parts of a standard Boston Scientific implanted device (Antoniadis et al., 2017). This virtual device had a non-septal RV lead, with a superior vena cava (SVC) coil in the right atrium (RA), and a straight LV lead through the coronary sinus, with four sensing LV tips distanced equally at 7.5mm. Configurations of both 12-lead ECG and CIED are shown in Figures 4A–D. All sensing electrodes, including the can of the implanted device (CAN), were approximated to single points, to increase the speed of LF computations and subsequent simulations. The computation of these matrices was only performed once for each torso configuration and took ∼8 min (128 cores). Their combination with the RE solutions produced high-fidelity 12-lead ECGs and EGM traces (Figures 4E,F) in ∼20 s (256 cores) for each paced location. Eight EGM vectors were chosen as the main EGM signals (Monaci et al., 2020), and included far-field CAN-SVC, CAN-RV, and SVC-RV, and near-field RV tip-RV ring and each LV tip-RV tip. However, importantly, additional vector combinations (four for ECGs and eight for EGMs) were added to the standard signals to facilitate integration into the CNN algorithms (see section “CNNs Training and Testing”).

**FIGURE 5:**
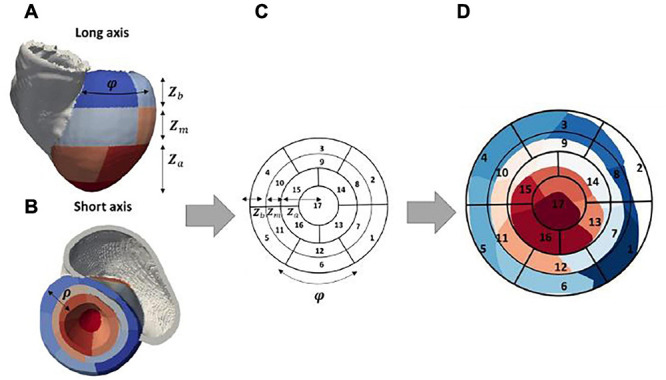
Patient-specific bull’s-eye diagram. 3D patient-specific 17-segment model in **(A,B)** can be related to the 2D representation in **(C)** by considering UVC coordinates *φ* and *z*. Specifically, *z* (the distance from apex to base) can be linked to the radius of the 2D diagram, as shown, separately for each apical (Za), mid (Zm), and basal (Zb) part of the model. *φ* is linked to the segments along the spherical axis. The final patient-specific 2D 17-segment model can be seen in **(D)**, where the various segments (1–17) are illustrated from blue-to-red color range [mapped from panel **B**].

Finally, a standardized universal ventricular coordinate (UVC) system was computed on the biventricular mesh (Bayer et al., 2018) to facilitate the development of a novel CNN specific to the ventricles, which should be advantageous as it identifies and constrains the localization of the paced beats inherently within the myocardium. As shown in Figure 5, UVCs describe the biventricular mesh using three parameters: z—normalized distance between apex (0) and base (1) along the long axis, ρ —normalized distance between endocardial (0) and epicardial (1) surfaces along the short axis, and φ —rotational distance from LV septum.

### CNN Architectures

In this study, we developed two separate 2D CNN architectures, which used the same ECG and EGM traces as inputs to identify the location of a simulated paced beat (representing an ectopic VT). The first architecture, based on Yang et al. (2018), locates the origin of the paced beat in Cartesian coordinates, after converting the outputs of the CNNs. The second utilizes a regression and a classification CNN to locate the VT in UVC space, naturally constraining the final localization of the focal VT source to the myocardium.

The existing Cartesian probability-based architecture was reproduced from Yang et al. (2018) and is composed of two classification CNNs named *Segment* and *EpiEndo* CNNs. *Segment* CNN classified in which LV segment the pacing beat originated, whereas *EpiEndo* CNN determined whether the pacing was endocardial or epicardial (binary decision). In our study, we developed two separate *Segment* CNNs: one classified between 17 LV segments (CNN with 17 output neurons) and the other between 68 LV segments (68 output neurons). Briefly, the structure of both *Segment* and *EpiEndo* CNNs consisted of two hidden layers alternating with two pooling layers and terminating with a fully connected (FC) layer. The output of both *Segment* and *EpiEndo* CNNs was a probability distribution (likelihood of each output neuron being the correct class). These distributions were obtained utilizing a softmax function on the output of the final FC layer. As performed in Yang et al. (2018), the probability distributions $(P_{seg}^{i}$ and $P_{epiendo}^{j}$ for *Segment* and *EpiEndo*, respectively) of each output segment (largest probability) and its adjacent segments were then combined with the centers of gravity *CoG(x, y, z)ij* of the corresponding endocardial and epicardial surfaces, as shown in Equation 1, to localize a paced beat in Cartesian coordinates.

(1) $${\left(S_{cartesian}\right)}_{out}=\sum_{i=1}^{N}P_{seg}^{i}\times \left(\sum_{j=1}^{2}P_{epiendo}^{j}\times CoG\left(x,y,z\right)ij\right)$$

The distance of the localized sources to the ground truths (simulated sources) was expressed in terms of localization errors (computed as Euclidean distance in millimeters).

**FIGURE 6:**
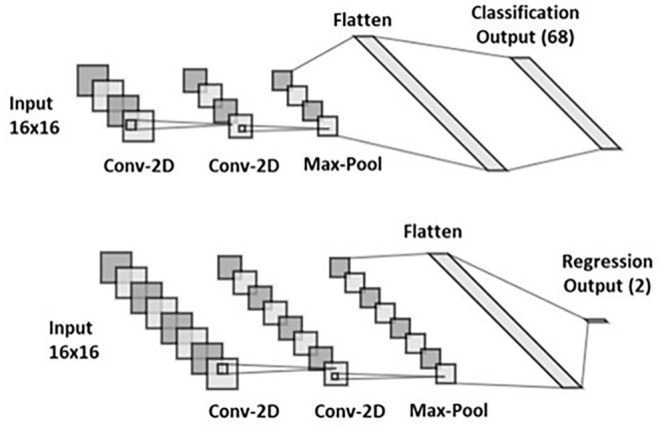
UVC-based convolutional neural networks (CNNs). Structure of 68-feature classification (top) and 2-output regression (bottom).

Our novel UVC-based algorithm is composed of one regression CNN, outputting *z* and ρ, and one 68-feature classification CNN, predicting the rotational coordinate φ. The structure of both CNNs was similar to the Cartesian probability-based network (hidden layer–hidden layer–pooling layer–FC layer), as shown in Figure 6. Because of the cyclic nature of φ, a three-output regression would have not returned satisfying and accurate results; hence, we used φ to divide the LV into 68 “wedges” (φ was grouped into intervals of 0.09 radians with each class assigned a label from 1 to 68, spanning φ = −π to φ=π). Using a higher number of LV “wedges” would have not returned desirable accuracies; thus, we decided to use a number of features (68) that had worked for *Segment* CNN and that was still suitable for achieving precise localization along *φ*. For the final localization of the paced beats, the outputs of the 68-feature classification (“wedges” with the highest probabilities) were converted back to φ, and combined with *z* and ρ regression predictions.

Both Cartesian probability-based and UVC-based algorithms were implemented in Python using Scikit-learn (Pedregosa et al., 2011) and TensorFlow (Abadi et al., 2015).

### CNN Localization Performance

Localization performance of the Cartesian probability-based algorithm was expressed in terms of localization error in millimeters, as described above. The same metric was used for our novel UVC-based architecture; however, the predicted values were first transformed from UVC space to Cartesian space [by locating the closest node in the mesh, with appropriate scaling of UVC coordinates (Bayer et al., 2018)] and then the distance with the ground truths (simulated pace beats) was evaluated (Euclidian distance, in millimeters).

For the *Segment* CNN of the Cartesian probability-based architecture, testing performance was evaluated in terms of accuracy, to allow comparison with results from Yang et al. (2018). Accuracy is defined as the percentage of paced beats correctly classified within each segment.

### CNNs Training and Testing

Training and testing inputs of both Cartesian probability-based and UVC-based CNNs were ECG and EGM traces computed from 3767 pacing excitations across the LV. To facilitate the execution of the 2D CNNs, the ECG and EGM signals had to be placed in square matrices; hence, we added four additional leads to the standard 12-lead ECGs [LL-RA-LA, LA-RA-LL, RA-LA-LL, and (RA+LA+LL)/2], as performed in Yang et al. (2018), and eight more EGM vectors to the standard eight EGMs (CAN-each LV tip and SVC-each LV tip), to achieve a total of 16 combinations of ECG and EGM vectors. QRSs were then extracted and sampled in time (16 time points), and stacked in 16 × 16 matrices, as shown in Figures 4E,F. A total of 2767 sets of these ECG and EGM matrices were used for training and were uniformly distributed across the myocardium (∼36% intramural/mid-wall, ∼32% epicardial, and ∼32% endocardial), with the exception of *EpiEndo* training data, which were epicardial and endocardial only (Yang et al., 2018). White Gaussian noise with a signal-to-noise ratio (SNR) of 25 dB was then added 10 consecutive times to all 16 ECG and EGM leads of the training set to augment the data by 10-fold (∼27,670) and increase robustness of the CNN training. A 10-fold cross-validation was performed in the existing Cartesian probability-based CNNs as part of the training (Yang et al., 2018), with a 90% (training)–10% (validation/testing) split. On the other hand, the cross-validation was used for hyper-parameter tuning in the UVC-based networks. After training, the localization performance of both Cartesian probability-based and UVC-based networks was tested by feeding the retained 1000 sets of ECG and EGM QRSs, with a SNR of 25 dB. Parameters of both *Segment* and *EpiEndo* CNNs were taken from Yang et al. (2018); batch size was set to 23, number of epochs was set to 10, learning rate was set to 0.001, and cross-entropy was used as loss function. A ReLU function was used as the activation function for feed-forward propagation, and a gradient-descent-projection method was used as the back propagation algorithm. In our UVC-based networks, we used similar parameters, except for the regression where we set the batch size to 23 and the number of epochs to 15, and we used mean absolute error as loss function.

### Investigation of Model Uncertainties

**FIGURE 7:**
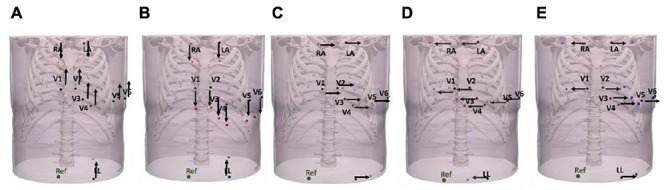
Variations in ECG electrode placements. ECG leads were displaced by ∼5 cm upward **(A)**, downward **(B)**, toward the left **(C)**, toward the right **(D)**, and by ∼10 cm (mixed displacements toward the right and left) **(E)**.

**FIGURE 8:**
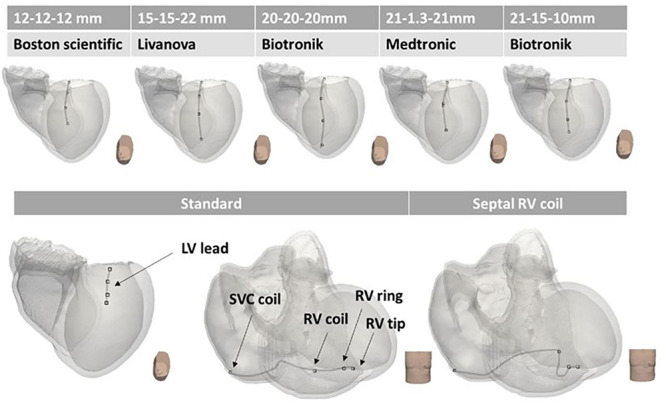
Variations in CIED configurations. Different CIED LV configurations according to different manufacturers (Boston Scientific, Livanova, Biotronik, and Medtronic) are shown on the top panels. The standard configuration of RV and LV leads is illustrated on the bottom left, and the septal RV coil configuration is shown on the bottom right. The main difference between the various configurations is the inter-electrode distance (reported above the manufacturers’ names).

Localization performance of both Cartesian probability-based and UVC-based CNN architectures, trained on the data described above, was also investigated by introducing different noise levels to the retained 1000 sets (SNR = 5, 10, 15, 20, and 30 dB). Moreover, we investigated the localization performance of both architectures as body compositions of the torso model were also varied, shown in Table 2, as well as different ECG electrode configurations (Figure 7) and different CIED configurations (Figure 8). For all these variations, LF matrices were recomputed (according to the new organ conductivities or electrodes positions) and combined with the retained 1000 intramural excitations to obtain new ECG and EGM matrices. These traces were then used to test both previously trained CNN architectures. Some of the major organ conductivities were varied according to physiological variations (Trakic et al., 2010; Sovilj et al., 2014); however, we chose to pair specific changes (for instance, liver and lungs, fat/muscle, named “bath” and liver, and different blood pools, etc.) to challenge CNN localization performance. ECG electrodes were displaced by 5 cm in all major orthogonal directions, and across all leads. Specifically, we shifted all ECGs leads upward (Figure 7A) and downward (Figure 7B)—RA and LA were always shifted downward, and LL upward—toward the left (Figure 7C) and the right (Figure 7D). Moreover, in one configuration (Figure 7E), the distance between ECG leads was increased by ∼10 cm. Finally, we simulated variations in electrode location and diameter of the virtual implanted device, as reported in Antoniadis et al. (2017) for different cardiac-resynchronization therapy (CRT-D) devices available in the market. Specifically, we changed the spacing between the sensing electrodes of the straight LV lead, to account for shorter or longer inter-electrode distance; in addition, we increased the diameter of RV and LV tips to ∼2 mm. In the latter scenario, instead of considering the EGM signals from single point electrodes, we averaged the signals obtained from a cloud of points within a 2-mm radius, to simulate more realistic conditions, and investigate whether our single point approximation of the CIED leads could affect the final localization. Finally, we considered the case of a septal RV coil configuration, which has been tested in CRT-D (Leclercq et al., 2016) and cardioverter defibrillators (ICD) (Winter et al., 1998).

**TABLE 2:** Variations in body compositions.

(1) Liver: 0.023 (S/m) and lungs: 0.039 (S/m)
(2) Bath: 0.45 (S/m) (pure muscle) and lungs: 0.039 (S/m)
(3) Bath: 0.05 (S/m) (pure fat) and lungs: 0.203
(4) Liver: 0.2 (S/m) and lungs: 0.039 (S/m)
(5) Bath: 0.05 (S/m) and lungs: 0.039 (S/m)
(6) Bath: 0.45 (S/m) and lungs: 0.203 (S/m)
(7) Bath (all organs except lungs): 0.24 (S/m) and lungs: 0.07 (S/m)
(8) Skin: 0.117 (S/m)
(9) Atria, ventricles, and aorta: 0.84 (S/m)

*Combinations of different organ conductivities within physiological changes are here reported, used in our CNNs sensitivity analysis.*

## Results

### Utility of EGMs in Existing Segment/EpiEndo-Based CNNs

**FIGURE 9:**
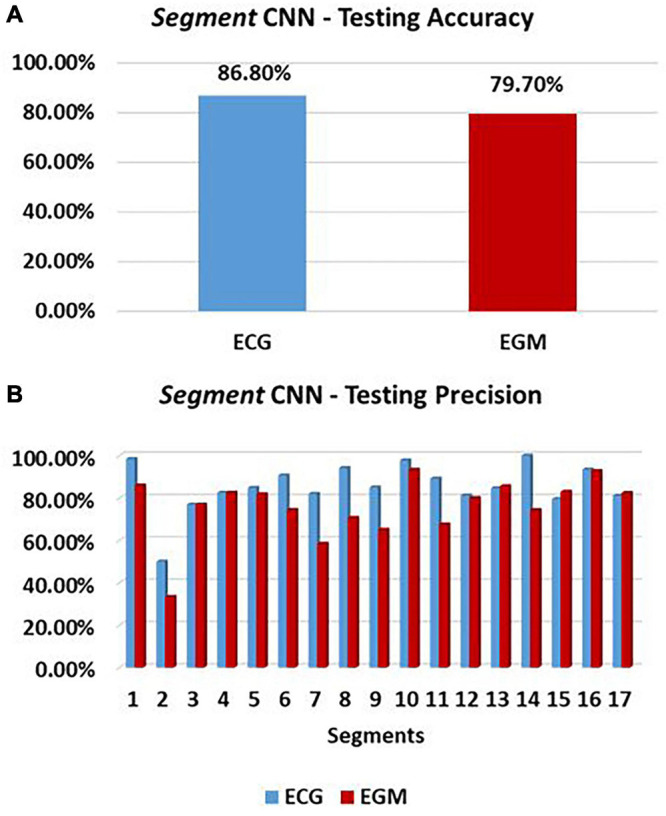
Cartesian probability-based CNN performance. Testing performance of 17-feature Segment CNN is here reported in terms of accuracy (%) **(A)**, and precision **(B)** for each of the 17 segments of the LV mesh. ECG-based and EGM-based testing performances are reported in blue and red, respectively.

We successfully reproduced the existing classification CNNs, namely, *Segment* and *EpiEndo*, introduced by Yang et al. (2018), to be trained and tested not only on ECG traces, but also on 16 different combinations of EGM vectors from a standard CIED with RV and LV leads. Testing performance of *Segment* CNN was similar for both ECG-based and EGM-based testing. As shown in Figure 9A, 86.76% accuracy was achieved for ECG-based testing and 79.70% was achieved for EGM-based testing (SNR = 25 dB).

The precision of *Segment* CNN in each segment, which defined how correct the CNN is at classifying one segment, is shown in Figure 9B. Here, we see that ECGs and EGMs have a similar influence on the network in almost every segment, with only few exceptions. The three highest precisions are in segments 1, 10, and 14 for ECG-based testing, and 1, 10, and 16 for EGM-based testing. The three lowest are in 2, 3, and 15 for ECGs, and 2, 7, and 9 for EGMs.

### Utility of EGMs in Cartesian Probability-Based Localization

**FIGURE 10:**
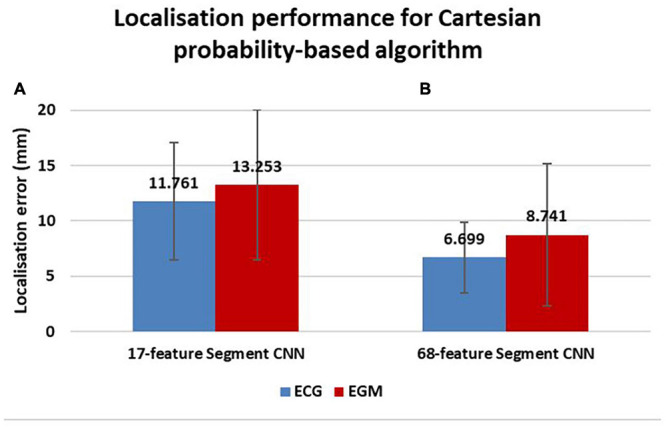
Localization performance of Cartesian probability-based algorithm. Localization errors in millimeters are reported for ECG-based (blue) and EGM-based (red) testing. A comparison in localization performance between different *Segment* CNNs can also be seen; the 17-feature *Segment* CNN is on the left **(A)** and the 68-feature *Segment* CNN is on the right **(B)**.

Localization in Cartesian space of each paced beat, from either ECG or EGM signals, was possible by combining probability distributions of *Segment* and *EpiEndo* CNNs (as shown in Equation 1). The localization performance, defined as the Euclidean distance in millimeters between an estimated source and the real location of the simulated paced beat, for the testing dataset of 1000 cases, is reported in Figure 10 for ECG-based and EGM-based testing. ECG-based localization and EGM-based localization produced a mean localization error of 11.76 ± 5.32 mm and 13.25 ± 6.79 mm, respectively.

Application of the 68-feature *Segment* CNN, based on the 68-segment LV AHA model shown in Figure 2G, was able to reduce localization errors of both ECGs and EGMs to 6.69 ± 3.19 mm and 8.74 ± 6.41 mm, respectively, as shown in Figure 10.

### UVC-Based Localization

**FIGURE 11:**
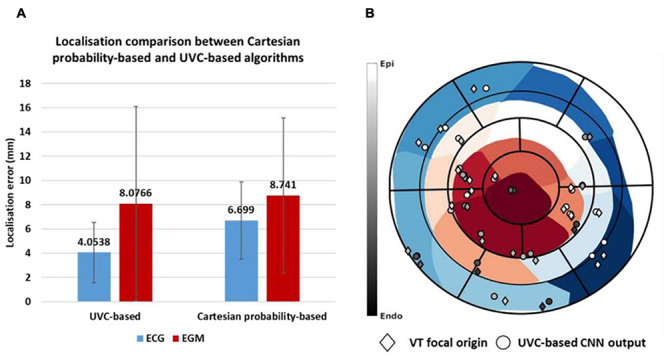
Localization comparison between UVC-based and Cartesian probability-based algorithms. Mean localization errors **(A)** are reported in millimeters with corresponding standard deviations for ECG-based (blue) and EGM-based (red) testing. An example of how **ventricular tachycardia** (VT) focal origins compare to UVC-based localized sources is shown in **(B)**; diamonds represent the ground truths, whereas the circles are the CNN outputs. The gray color bar represents the distance from endocardial (black) to epicardial (white) surfaces of each source, whereas the blue-to-red color bar represents 1–17 patient-specific AHA segments.

Further improvements in localization performance were made by developing two CNNs, which returned the position of a paced beat in a reference frame specific to the ventricles (UVCs). This UVC-based localization outperformed the Cartesian probability-based localization, as shown in Figure 11A, reducing localization errors to 4.06 ± 2.47 mm and 8.07 ± 8.26 mm for ECG and EGM-based testing, respectively.

UVC-based localized sources are visualized in 2D in a patient-specific bull’s-eye diagram, shown in Figure 11B for 30 beats, as previously illustrated in Figure 5. Here, a paced beat can be visualized using its UVC coordinates and can be compared to the ground truth, revealing a close match between all pairs. The radius of the diagram describes the distance of a paced beat to the LV apex (center of the diagram), relatable to UVC *z*, and its circumferential direction (*φ*) facilitates the positioning of the beat within a specific segment. The intramural location (*ρ*) of the beat (how far from the endocardium and/or epicardium) is color coded.

### Sensitivity to Noise

**FIGURE 12:**
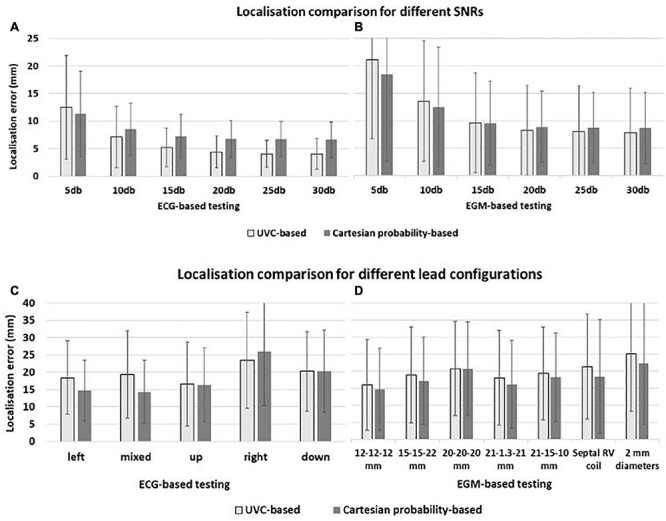
Localization comparison between UVC-based (light gray) and Cartesian probability-based (dark gray) networks. Localization performance for ECG-based and EGM-based testing are here reported during sensitivity analysis for different noise levels—**(A,B)**, respectively—and for different lead configurations—**(C,D)**, respectively. For all panels, mean errors with corresponding standard deviations are reported for UVC-based (light gray) and Cartesian probability-based (dark gray) localization. Displacements of ECG leads, shown in Figure 7, results in localization errors in **(C)**; on the other hand, different CIED configurations, shown in Figure 8, return errors in **(D)**. Little difference in localization is present between the two networks with similar mean localization errors.

Overall, localization was only slightly affected by noise, as seen in Figure 12A (ECG-based localization) and Figure 12B (EGM-based localization). As SNR decreased (increased noise), localization errors increased only slightly, with one exception (SNR = 5 dB), where the performance of both UVC-based and Cartesian probability-based localization was reduced. However, all localization errors were < 12.5 mm for ECG-based localization. Moreover, noise seemed to affect EGM-based localization more than ECG-based localization.

### Sensitivity to Electrode Locations

Displacements of ECG leads and different CIED configurations did affect the localization performance of UVC-based and Cartesian probability-based algorithms (errors > ∼15 mm) for ECG- and EGM-based testing, as shown in Figures 12C,D, respectively. ECG-based localization was more affected by displacements away from the heart (20 mm)—right and downward shifts. Errors in EGM-based localization were higher (20 mm) when considering longer inter-electrode distance (20 mm) and increased lead surface diameter (2 mm). For UVC-based localization, a septal RV coil configuration caused errors to increase > 20 mm as well.

### Sensitivity to Tissue Conductivities in Torso Model

**FIGURE 13:**
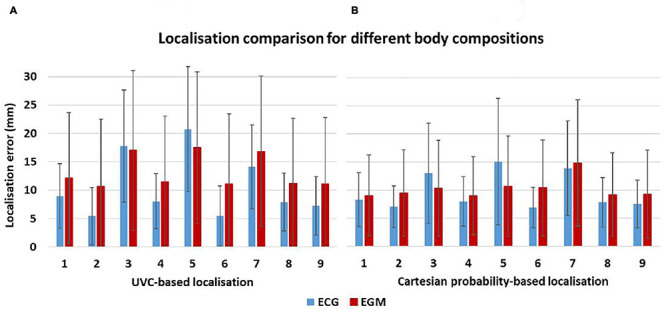
Localization sensitivity to tissue conductivities. Mean localization errors are here reported for UVC-based **(A)** and Cartesian probability-based **(B)** localization as different organ conductivities were changed in the torso model—see Table 2. Small differences are seen between ECG-based (red) and EGM-based (blue) localization errors.

A comparison between ECG-based and EGM-based localization for different body compositions is shown in Figure 13A (for UVC-based localization) and Figure 13B (for Cartesian probability-based localization). ECG-based localization was only affected by a high increase of fat in the torso bath (scenarios 3 and 5) and when the whole torso was simplified to bath and lungs (scenario 7). In those three scenarios, mean localization errors increased to 17.75 ± 9.88 mm, 20.72 ± 10.99 mm, and 14.08 ± 7.38 mm for UVC-based testing, respectively, and to 13.01 ± 8.89 mm, 15.07 ± 11.20 mm, and 13.88 ± 8.40 mm for 68-segment Cartesian probability-based testing. Other variations of tissue conductivity did not affect the performance of either algorithm (localization errors < ∼8 mm).

Finally, EGM-based testing was less affected by changes in body compositions during Cartesian probability-based localization. Similar to above for ECG-based localization, simplification of the torso to bath and lungs (scenario 7) caused the highest mean error (14.83 ± 11.24 mm), but in all other scenarios, localization ranged between 9.04 and 10.66 mm. On the other hand, UVC-based EGM localization had a similar trend to ECG-based localizations, with errors > 15 mm for scenarios 3, 5, and 7.

## Discussion

In this study, we successfully utilized simulated implanted device EGMs to localize virtual focal VT sources using CNNs, achieving accuracies that could be useful in clinical settings. A previous algorithm (Yang et al., 2018) utilized 12-lead ECGs for a similar purpose; here, we managed to replicate the structure of the CNN architecture for EGM traces and improve the overall localization by introducing a higher number of segments in the AHA LV model. Moreover, we also improved the overall localization precision by introducing a novel architecture composed of regression and classification algorithms, which was able to identify the source in a framework specific to the ventricles, easily interpretable by clinicians. Finally, we investigated the robustness of both CNN algorithms to the introduction of uncertainties, such as different noise levels in the data, and possible inter-patient variabilities (different body compositions, ECG lead positions, and CIED configurations).

### Successful Application of EGMs in Existing Cartesian Probability-Based Algorithm

Simulated focal VT sources were successfully identified from 16 combinations of implanted device EGM vectors. In the previous study, Yang et al. (2018) achieved localization precision in the range of 10–11 mm when utilizing a combination of 16 ECG vectors; here, we reduced localization errors to 6.69 mm and 8.74 mm for ECG and EGM traces, respectively, by incrementing the number of segments in the LV to 68. In clinical practice, the average diameters of catheter tips are between 4 mm and 8 mm (Ilg et al., 2010), limiting the average lesion size to a minimum of ∼8.5 mm (Wittkampf et al., 1989). Hence, we achieved localization precisions in a range suitable for improving ablation planning. Especially in patients with a non-stable condition, pre-planning of these procedures could be expedited and aided if the acquisition of ECG data during VT would not be required, which can be achieved by utilizing information stored in implanted devices. Our algorithm thus proposes a first level of investigation that could direct clinicians to the region of interest with high precision. Moreover, we achieved ECG-based and EGM-based testing accuracies in ranges comparable to Yang et al. (2018) (77%). Similar patterns to the previous study were seen when investigating how noise affects the localization; only a loss in accuracy and localization precision is seen with SNR = 5 dB. Interestingly, noise seems to have a greater impact on EGM-based localization than on ECGs. This could be explained by the fact that implanted device sensing vectors are closer to one another and to the cardiac electrical activity, amplifying variations caused by noise, thus affecting EGM-based localization to a greater degree.

### Novel UVC-Based Algorithm Improves Localization

Our novel UVC-based algorithm improves localization to 4.06 mm and 8.07 mm for ECGs and EGMs, respectively, outperforming the existing study. Whereas the Cartesian probability-based algorithm relies on combining probabilities of two networks with the geometrical centers of gravity of each LV segment to locate a VT origin, our architecture predicts the actual location of the source in terms of its (normalized) distance from the apex, the LV septum and, most importantly, the endocardium. Furthermore, it intrinsically bounds the localization to the myocardium. Knowing the exact intramural (mid-wall) location, a VT source could help in the choice of power, tip diameter, and lesion size to apply, as well as access direction (epicardial or endocardial), in pre-procedural planning. Finally, our novel localization facilitates the visualization of focal estimates, by plotting a patient-specific bull’s-eye diagram, where the radius represents the distance from the apex and the circumferential direction relates to septal, anterior, inferior, and lateral LV segments.

### Automated Localization Is Only Affected by Extreme Changes in ECG Lead Positions and Implanted Device Lead Configurations

Focal VT localization is only marginally affected by differences in body compositions. However, to increase the accuracy of the results, a torso model constructed for algorithm training should at least include all major thoracic and abdominal organs with realistic conductivity values; our findings suggest that representing bath and lungs (as used in Yang et al) only produce signals that differ substantially from more complete torso models, importantly affecting localization accuracy. Moreover, EGM-based algorithms seem to be more robust to tissue variations, possibly due to the closer positioning of the device leads to the electrical cardiac source, with extracellular potentials being less affected by the surrounding tissue/organ conductivities. Displacements in ECG leads and differences in common CIED configurations do seem to have an impact on the final localization; this suggests the necessity of integrating a higher variability in the training data, or extrapolating ECG or CIED patient-specific information from imaging data to strengthen future automated algorithms and allow clinical validation and translation.

### Limitations

A notable limitation of this study is the absence of clinical validation. However, our main goal was to strengthen the automated localization of focal VTs and investigate the properties of our 3D pipeline that need improvement for future clinical studies. For future validation of our *in silico* EGM model and corresponding CNN localization, we will need to generate patient-specific 3D models that have been registered and tuned to the clinical framework used during EP mapping and ablation, collect simulated data on such models for CNN training, and test the latter on clinical EGM recordings of the focal VT(s), and/or paced beats, that have been collected from CIEDs directly or from the latter recording during the mapping. When attempting clinical translation in the future, we will also investigate other aspects of our work regarding patient-specific EP properties that were not taken into consideration in this study. Our model required certain simplifications, such as rule-based fibers and lack of Purkinje activation, which we believe would not make an impact in the final performance of our algorithms when dealing with focal beats, but that could be useful to take into account for more complex patient-specific approaches. Although our cardiac model was static, we do not believe that the absence of electro-mechanical feedback significantly influenced the final ECG or EGM signals, when considering only QRSs (ventricular activation); many studies have validated static simulated EGM signals against clinical data (Cardone-Noott et al., 2016; Cedilnik et al., 2018; Gillette et al., 2021), showing that it is not necessary to couple mechanical simulations with EP for these types of problems. Moreover, we only considered single beats originating in the LV. In future studies, it will be worth including focal VTs in the RV, which is a common region of VT especially around the outflow track (RVOT). This could be easily achieved by using the UVC system, which covers the RV, to generate labels and prepare simulations, facilitating both modeling and localization pipelines. Although we believe that simulating multiple paced beats would not have an impact in the final CNN performance and localization, it will be necessary to achieve more realistic scenarios, as it can influence the waveforms of ECG and EGM traces. Furthermore, extending the automated localization of VT to more complex episodes (for instance, in presence of micro re-entries and/or infarction) represents an interest of ours that will be addressed in future studies. The investigation on how different signal uncertainties influence the performance of our CNNs could also be extended to include more complex and realistic ways of adding noise to customize computational models to patient-specific settings (Barone et al., 2020; Marcotte et al., 2021). Another aspect of this study that could be refined is the overall structure of our novel UVC-based architecture; both regression and classification networks were implemented following the structure proposed by Yang et al. (2018), although some parameters were optimized to fit the new tasks. In future studies, deeper networks could be developed, and different input data shapes could be investigated (e.g., 2D vs. 1D). Moreover, to tackle the problem of computational efficiency and decrease even further our simulation time when dealing with more complex arrhythmias, we may investigate the possibility of GPU-based models, which have recently opened new perspectives in terms of real-time, physiologically detailed simulations (Vasconcellos et al., 2020).

## Conclusion

By integrating fast EP simulations with deep-learning algorithms, we have demonstrated the utility of our *in silico* pipeline for the simulations of EGMs stored in implanted devices, which, in addition to 12-lead ECGs, can accurately localize focal VTs. Our novel *in silico* automated algorithm, which utilizes a coordinate frame specific to the ventricles, increased localization precision above previous segment-classification approaches, facilitating clinical interpretation. Moreover, we showed the necessity of including more variability in the training data regarding lead positions, and the stability, on the other hand, of the localization to changes in body compositions.

## Data Availability Statement

The raw data supporting the conclusions of this article will be made available by the authors, without undue reservation.

## Author Contributions

SM, KG, and EP-A were primarily led the work and consulted in the initial coding of lead field computations and CNN structure, respectively. RR provided the CT data used for torso model creation. GP was responsible for developing and providing the reaction-eikonal and lead field packages within CARP. Finally, AK and MB supervised the project, guided the study design, and provided useful insights into and feedback on every aspect of the work (from the modeling side to the localization pipeline and the CNN algorithms). All authors contributed to the article and approved the submitted version.

## Disclaimer

The views expressed are those of the author(s) and not necessarily those of the National Health Service, the National Institute for Health Research, or the Department of Health.

## Conflict of Interest

The authors declare that the research was conducted in the absence of any commercial or financial relationships that could be construed as a potential conflict of interest.
